# Pollen morphology and variability of Polish native species from genus *Salix* L.

**DOI:** 10.1371/journal.pone.0243993

**Published:** 2021-02-18

**Authors:** Irmina Maciejewska-Rutkowska, Jan Bocianowski, Dorota Wrońska-Pilarek

**Affiliations:** 1 Department of Forest Botany, Poznań University of Life Sciences, Poznań, Poland; 2 Department of Mathematical and Statistical Methods, Poznań University of Life Sciences, Poznań, Poland; Georg-August-Universitat Gottingen, GERMANY

## Abstract

The pollen morphology was studied of 24 *Salix* species native to Poland, which represented two subgenera, 17 sections and five subsections occurring in Poland. The aim of this study was to discover the taxonomical usefulness of the pollen features under analysis, and to investigate the ranges of their interspecific variability. In total, 720 pollen grains were studied. They were analysed with respect to seven quantitative features (length of the polar axis ˗ P, equatorial diameter ˗ E, length of the ectoaperture ˗ Le, exine thickness ˗ Ex, and P/E, Ex/P and Le/P ratios) and the following qualitative ones: pollen outline and exine ornamentation. The most important features were exine ornamentation (muri, lumina and margo) characters. The pollen features should be treated as auxiliary because they allowed to distinguish eight individual *Salix* species, and five groups of species. Statistical analysis of the studied traits indicated a high variability among the tested species. The most variable biometric features were P, E and Le, while lower variability occurred in P/E, Le/P and d/E.

## Introduction

The genus *Salix* L. (Salicaceae) consists of deciduous (and, rarely, semi-evergreen) trees and shrubs, including dwarf forms, with decumbent shoots, mainly distributed across the cold and moderate climate zones of the Northern Hemisphere. The number of *Salix* species is estimated to be from 330 up to even 530 worldwide, with the highest species concentration in northern Eurasia, northern North America, and in the mountains of China [1–5]. There are 65 willow species described in Europe, including 27 which are native to Poland [6, 7]. Following the generic and subgeneric treatments of Euroasian *Salix* taxa proposed by Skvortsov [3], the Polish species represent three subgenera, 17 sections and five subsections.

*Salix* is considered one of the most taxonomically difficult plant genera and its infrageneric taxonomy is still in progress. It is a result of, among others, a very simplified and undifferentiated flower structure, which limits the use of generative traits in *Salix* systematics. All willow species are dioecious, and their flowers and leaves usually develop at different times. Therefore, field observations of an individual plant are rather inconvenient. Many *Salix* species exhibit significant morphological variations, correlated with high infraspecific genotypical polymorphisms. This is often reflected in intra-species division into numerous subspecies, varieties and forms. At the same time, the differences between some *Salix* species are difficult to define [3, 5, 8]. Willows are also highly cross-compatible, and numerous hybrids have been recognised, both natural and artificial. It is difficult to estimate the number of natural hybrids in Europe, or even in certain regions of the continent. Meikle [9] and Rechinger [10] believed that the formation of spontaneous hybrids between *Salix* species within Great Britain was a frequent phenomenon. Field observations in transect from Greece to arctic Norway recorded a total of 20 willow species, along with 12 hybrids [11]. Similarly field and herbarium analyses of the occurrence of willow species in the whole Latvia revealed as many as 68 hybrids and 20 „pure” species of *Salix* [12]. In turn, Oberprieler et al. [13] provided molecular and phytochemical evidence *S*. ×*rubens* to be natural hybrid between *Salix alba* and *S*. *fragilis*.

The long history of *Salix* cultivation has resulted in the selection of over 850 cultivars, of which 734 are accepted by the International Poplar Commission [14].

The factors mentioned above contribute to the fact that not many researchers have undertaken extensive palynological investigations of this genus. Most of the palynological studies of *Salix* have focused on a specified geographical region with a limited number of species, up to 10 (compare [15–26] and others). One of the exceptions was Kim and Zsuffa’s elaboration [27], describing the pollen morphology of 15 Korean species, two varieties and one form, representing six sections of the subgenus *Salix* L. Later, Sohma [28] examined the pollen grains of 72 Asian *Salix* taxa. He noted certain differences in the exine patterns and, based on these differences, described eight types of exine ornamentation. The “Pal dat” database, established by Diethart and Halbritter [29–31], contains brief descriptions of 12 *Salix* species.

Knowledge of the morphological structure of *Salix* pollen grains is incomplete not only due to the limited number of species analysed. Researchers usually limit their analyses to individual and/or the most important pollen grain features (mainly pollen size and shape, or exine ornamentation). As yet, save the study by Kim et al. [22], no other research on the interspecific variability of willow species has been undertaken.

It was assumed that the examined pollen grain characteristics could help to identify the individual *Salix* taxa under analysis. Therefore, the main aim of this study was to discover the taxonomical usefulness of the quantitative and qualitative morphological features of pollen. The second goal was to describe, for the first time, the interspecific morphological variability of the pollen grains from the studied species of the genus *Salix*. It was assumed that the research results would be representative thanks to a complex comparative analysis of the diagnostic, morphological features of pollen from suitably selected plant material, representing all the intrageneric taxa distinguished at the present time (24 species from all subgenera, sections and subsections of willows found in Poland). Due to SEM observations detailed pollen morphology of the 6 studied species (*S*. *dasyclados*, *S*. *myrsinifolia*, *S*. *myrtilloides*, *S*. *rosmarinifolia*, *S*. *silesiaca*, *S*. *starkeana*) has not been described in the palynological literature so far.

## Material and methods

### Palynological analysis

The study was conducted on 24 of the 26 species of *Salix* native to Poland. The taxa under examination represented all three subgenera, all sections (17) and subsections (5) of willows found in Poland, including two legally protected species in Poland–*S*. *lapponum* and *S*. *myrtilloides* (collected from a specific herbarium). A list of the species analysed with their affiliation to particular taxa is shown in Table 1.

**Table 1:** List of localities with the taxonomic classification of the *Salix* species studied.

Subgenus *Salix*		
Sect. *Amygdalinae* Koch		
1.	*S*. *triandra* L.	Dendrological Garden, Poznań University of Life Sciences, 52°25′ N, 16°53′ E, 09.04.2019, I. Maciejewska-Rutkowska (POZNF 08/19)
Sect. *Pentandrae* (Borrer) Schneider		
2.	*S*. *pentandra* L.	Dendrological Garden, Poznań University of Life Sciences, 52°25′ N, 16°53′ E, 06.05.2019, I. Maciejewska-Rutkowska (POZNF 13/19)
Sect. *Salix*		
3.	*S*. *alba* L.	Dendrological Garden, Poznań University of Life Sciences, 52°25′ N, 16°53′ E, 23.04.2019, I. Maciejewska-Rutkowska (POZNF 12/19)
4.	*S*. *fragilis* L.	Dendrological Garden, Poznań University of Life Sciences, 52°25′ N, 16°53′ E, 14.04.2019, I. Maciejewska-Rutkowska (POZNF 11/19)
Subgenus *Chamaetia*/*Vetrix*		
Sect. *Chamaetia* Dum.		
5.	*S*. *reticulata* L.	Herbarium, Siwarowa Col under Wielka Turnia, Tatra Mts, 49°14′ N 19°54′ E, 16.08.1951, A. Jasiewicz (KRAM 416332)
Sect. *Retusae* Kerner		
6.	*S*. *retusa* L.	Botanical Garden, Adam Mickiewicz University in Poznań, 52°25 N 16°52′ E, 08.04.2019, I. Maciejewska-Rutkowska (POZNF 06/19)
Sect. *Myrtilloides* Koehne		
7.	*S*. *myrtilloides* L.	Herbarium,Totta, Khabarovskiy Kray, 57°74′ N 137°54 E, 11.06.1979, S. Kharkevich, T. Buch (KRAM 425601)
Sect. *Myrtosalix* Kerner		
8.	*S*. *alpina* L.	Herbarium, Giewont, Tatra Mts, 49°15′ N 19°55′ E, 06.1856, Berdan (KRAM 143849)
Sect. *Hastatae* Kerner		
9.	*S*. *hastata* L.	Herbarium, Hala Pyszna under Błyszcz ridge, Tatra Mts, 49°12′ N 19°51′ E, date unknown, M. Łańcucka (KRAM 219352)
Sect. *Nigricantes* Kerner		
10.	*S*. *myrsinifolia* Salisb.	Herbarium, Saransk, Republic of Moldovia, 54°11′ N 45°11′ E, 26.05.1971, KG. Maljtin (KRAM 363224)
Sect. *Vetrix* Dum.		
Subsect. *Vulpinae* Kimura		
11.	*S*. *silesiaca* Willd.	Botanical Garden, Adam Mickiewicz University in Poznań, 52°25 N 16°52′ E, 01.04.2019, I. Maciejewska-Rutkowska (POZNF 03/19)
Subsect. *Laeves* Camus		
12.	*S*. *caprea* L.	Dendrological Garden, Poznań University of Life Sciences, 52°25′ N, 16°53′ E, 19.03.2019, I. Maciejewska-Rutkowska (POZNF 01/19)
13.	*S*. *cinerea* L.	Field, near Bagno Chlebowo reserve (Notecka forest), 52°43′ N 16°45′ E, 15.03.2017, I. Maciejewska-Rutkowska (POZNF 02/17)
14.	*S*. *aurita* L.	Field, near Rusałka lake, Poznań, 52°25′ N 16°52′ E, 25.03.2019, P. Kiciński, I. Maciejewska-Rutkowska (POZNF 02/19)
Subsect. *Substriatae* Görz.		
15.	*S*. *starkeana*Willd.	Herbarium, Romaszkovo village, Moscow region, 55°42′ N 36°58′ E, 10.05.1972, WW. Makarow (KRAM 215673)
Sect. *Arbuscella* Seringe ex Duby		
Subsect. *Bicolores* A. Skv.		
16.	*S*. *phylicifolia* L.	Herbarium, Säksjärvi village, 61° 25′ N 23° 44′ E, 21.05.1956, L. Korhonen (KRAM 065111)
Sect. *Vimen* Dum.		
17.	*S*. *viminalis* L.	Dendrological Garden, Poznań University of Life Sciences, 52°25′ N, 16°53′ E, 01.04.2019, I. Maciejewska-Rutkowska (POZNF 04/19)
18.	*S*. *dasyclados* Wimm.	Herbarium, Orawka village, near Czarna Orawa stream, 49°30′ N 19°43′ E, 05.08.1963, M. Guzikowska (KRAM 039567)
Sect. *Canae* Kerner.		
19.	*S*. *elaeagnos* Scop.	Herbarium, Poronin, gravel pit, 49°20′ N 20°00′, 10.05.1952, Sławkowska, Sławkowski (KRAM 035666)
Sect. *Villosae* Rouy		
20.	*S*. *lapponum* L.	Herbarium, Štrbské Pleso, Tatra Mts, 49°07′ N 20°03′ E, 30.07.1933, V. Krajine (KRAM, 262753)
Sect. *Daphnella* Ser. ex Duby		
21.	*S*. *daphnoides* Vill.	Dendrological Garden, Poznań University of Life Sciences, 52°25′ N, 16°53′ E, 01.04.2014, I. Maciejewska-Rutkowska (POZNF 05/19)
Sect. *Incubaceae* Kerner		
22.	*S*. *repens* L.	Dendrological Garden, Poznań University of Life Sciences, 52°25′ N, 16°53′ E, 08.04.2019, I. Maciejewska-Rutkowska (POZNF 07/19)
23.	*S*. *rosmarinifolia* L.	Dendrological Garden, Poznań University of Life Sciences, 52°25′ N, 16°53′ E, 09.04.2019, I. Maciejewska-Rutkowska (POZNF 09/19)
Sect. *Helix* Dum.		
Subsect. *Purpureae* (Hayek) A. Skv.		
24.	*S*. *purpurea* L.	Dendrological Garden, Poznań University of Life Sciences, 52°25′ N, 16°53′ E, 09.04.2019, I. Maciejewska-Rutkowska (POZNF 10/19)

KRAM–herbarium of W. Szafer Institute of Botany, Polish Academy of Sciences, Kraków (Poland); POZNF–herbarium of Department of Forest Botany, Poznań University of Life Sciences (Poland).

The presented classification system of analysed *Salix* species was partly based on the current phylogenetic studies [32–34]. According to the molecular data, subgenus *Chamaeti*a and *Vetrix* are merged and subgenus *Chamaeti*a*/Vetrix* is accepted. Traditional division into the sections and subsections proposed by Skvortsov [3] is given, because it is practically the only infrageneric system that includes all 24 currently studied species of willows. Verification of the taxa was performed by taxonomist Prof. Jerzy Zieliński (Institute of Dendrology, Polish Academy of Sciences in Kórnik) [6].

Several, randomly selected inflorescences (flowers) were collected from the Dendrological Garden of UPP in Poznań, Several, randomly selected inflorescences (flowers) were collected from the Dendrological Garden of UPP in Poznań (- permission granted by the director, dr. T. Maliński), from the Botanical Garden of Poznań Adam Mickiewicz University (- permission granted by the director, professor J. Wiland-Szymańska), from the field within Wielkopolska region (- no permission required) and from a herbarium belonging to the Institute of Botany at the Polish Academy of Sciences in Kraków (-permission granted by the herbarium curator, dr. hab. Beata Paszko). All the plants grew on natural sites in Poland, Finland and Russia (Table 1). Five *Salix* species were collected outside Poland, due to the limited occurrence in the country. The plant material was stored in the herbarium of the Department of Forest Botany at the Poznań University of Life Sciences (POZNF). The authors unequivocally state that the material for the current research has been collected in accordance with the principles of ethics, without threatening the existence of the populations of the studied species in the future.

In accordance with the study by Wrońska-Pilarek et al. [35], each sample consisted of 30 randomly selected, mature and correctly formed pollen grains derived from a single individual (shrub or tree). In total, 720 pollen grains were studied.

The pollen grains were prepared for light (LM) and scanning electron microscopy (SEM) using the standard methods described by Erdtman [36]. The prepared material was divided into two parts: one part was immersed in an alcohol solution of glycerine (for LM) and the other in 96% ethyl alcohol (for SEM). Morphological observations were carried out using both a digital light microscope (Levenhuk D320L) and a scanning electron microscope (Jeol 7001TTLS). Eight quantitative features of the pollen grains were analysed, i.e. the length of the polar axis (P) and equatorial diameter (E), the length of the ectoaperture (Le), the exine thickness (Ex), and P/E, Le/P, Ex/P and Ex/E ratios. The pollen shape classes (P/E ratio) were adopted according to the classification proposed by Erdtman [15]: oblate-spheroidal (0.89–0.99), spheroidal (1.00), prolate-spheroidal (1.01–1.14), subprolate (1.15–1.33) and prolate (1.34–2.00). The following qualitative features were also analysed: the outline, shape and exine ornamentation.

Exine ornamentation types (1–8) were identified based on the classification proposed by Sohma [28]. The types of reticulate exine ornamentation were characterised by the height, width and course of the muri and diameter of the lumina.

The microphotographs of pollen grains of analysed *Salix* species were posted in GfBio (Submission ID: c3c22e7f-f0b9-43f5-a5d0-328399365b54).

The descriptive terminology follows Punt et al. [37] and Halbritter et al. [38].

### Statistical analysis

Firstly, the normality of the distributions of the studied traits (P, E, P/E, Le, Ex, Le/P, Ex/P and Ex/E) was tested using Shapiro-Wilk’s normality test [39]. A multivariate analysis of variance (MANOVA) was performed based on the following model using a MANOVA procedure in GenStat 18: Y = XT+E, where: Y is (*n*×*p*)–the dimensional matrix of observations, *n* is the total number of observations, *p* is the number of traits (in this study *p* = 8), X is (*n*×*k*)–the dimensional matrix of design, *k* is the number of species (in this study *k* = 24), T is (*k*×*p*)–the dimensional matrix of unknown effects, and E–is (*n*×*p*)–the dimensional matrix of residuals. Following this, one-way analyses of variance (ANOVA) were performed in order to verify the null-hypothesis of a lack of species effect, as opposed to the alternative hypothesis of significant differences among the species, in terms of the values of the observed traits, independently for each trait, based on the following model: *y*_*ij*_ = *μ*+*τ*_*i*_+*ε*_*ij*_, where: *y*_*ij*_ is *j*th observation of *i*th species, *μ* is the general mean, *τ*_*i*_ is the effect of *i*th species and *ε*_*ij*_ is an error observation. The minimal and maximal values of the traits as well as the arithmetic means and coefficients of variation (cv in %) were calculated. Moreover, Fisher’s least significant differences (LSDs) were estimated at a significance level of α = 0.001. The relationships between the observed traits were assessed based on Pearson’s correlation coefficients using a FCORRELATION procedure in GenStat 18. The results were also analysed using multivariate methods. Mahalanobis [40] distance was suggested as a measure of “polytrait” species similarity [40], the significance of which was verified by means of critical value D_α_ called “the least significant distance” [41]. The differences among the analysed species were verified by cluster analysis using the nearest neighbour method and Euclidean distances. All the analyses were conducted using the GenStat 18 statistical software package.

## Results

### General morphological description of pollen

**Fig 1:**
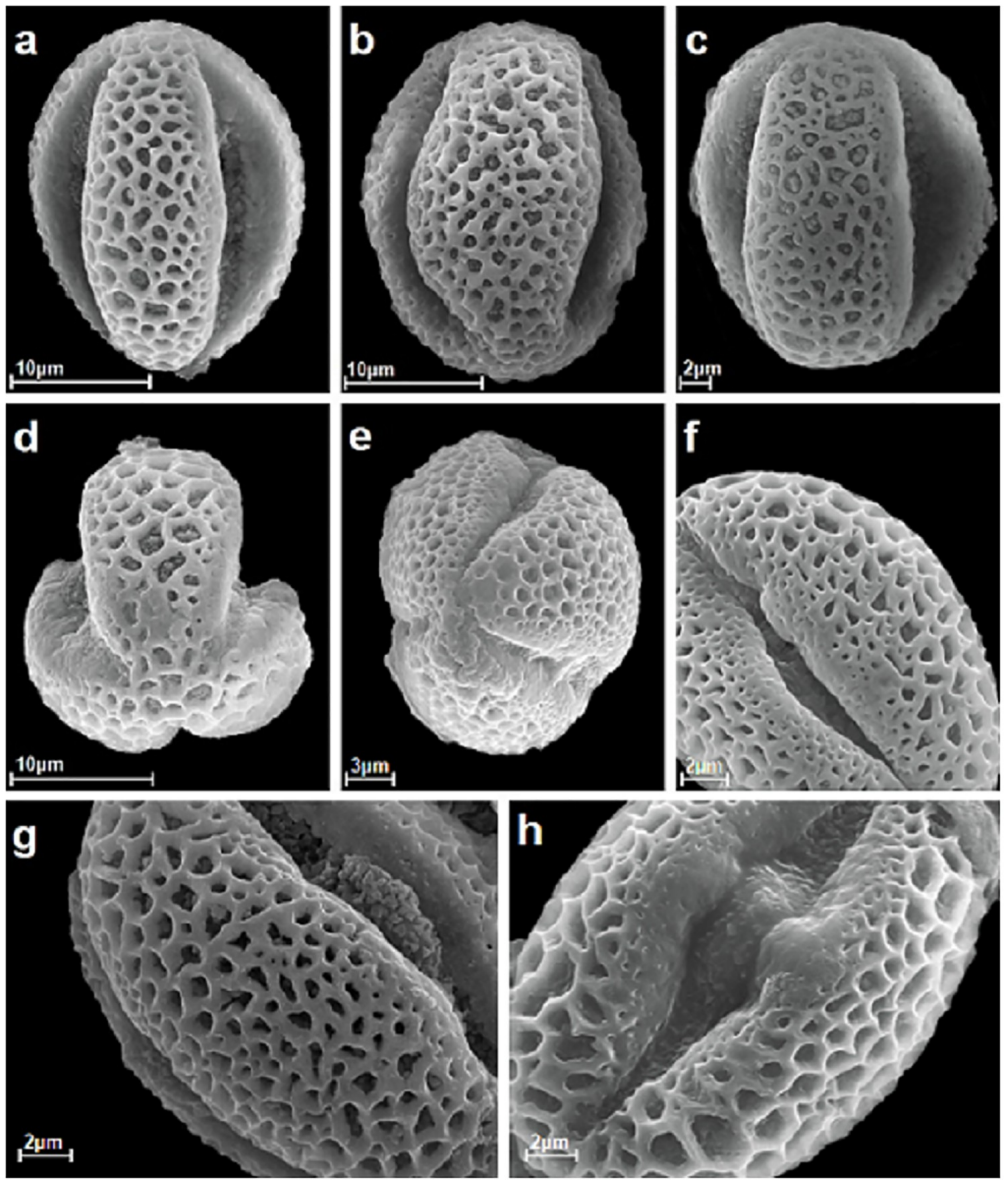
Pollen grains in equatorial view in *S*. *myrsinifolia*, *S*. *retusa*, *S*. *starkeana*, A-C; pollen grains in polar view in *S*. *myrsinifolia*, *S*. *triandra*, D-E; colpori with granulate membranes in *S*. *triandra*, *S*. *hastata*, *S*. *purpurea*, F-H.

**Fig 2:**
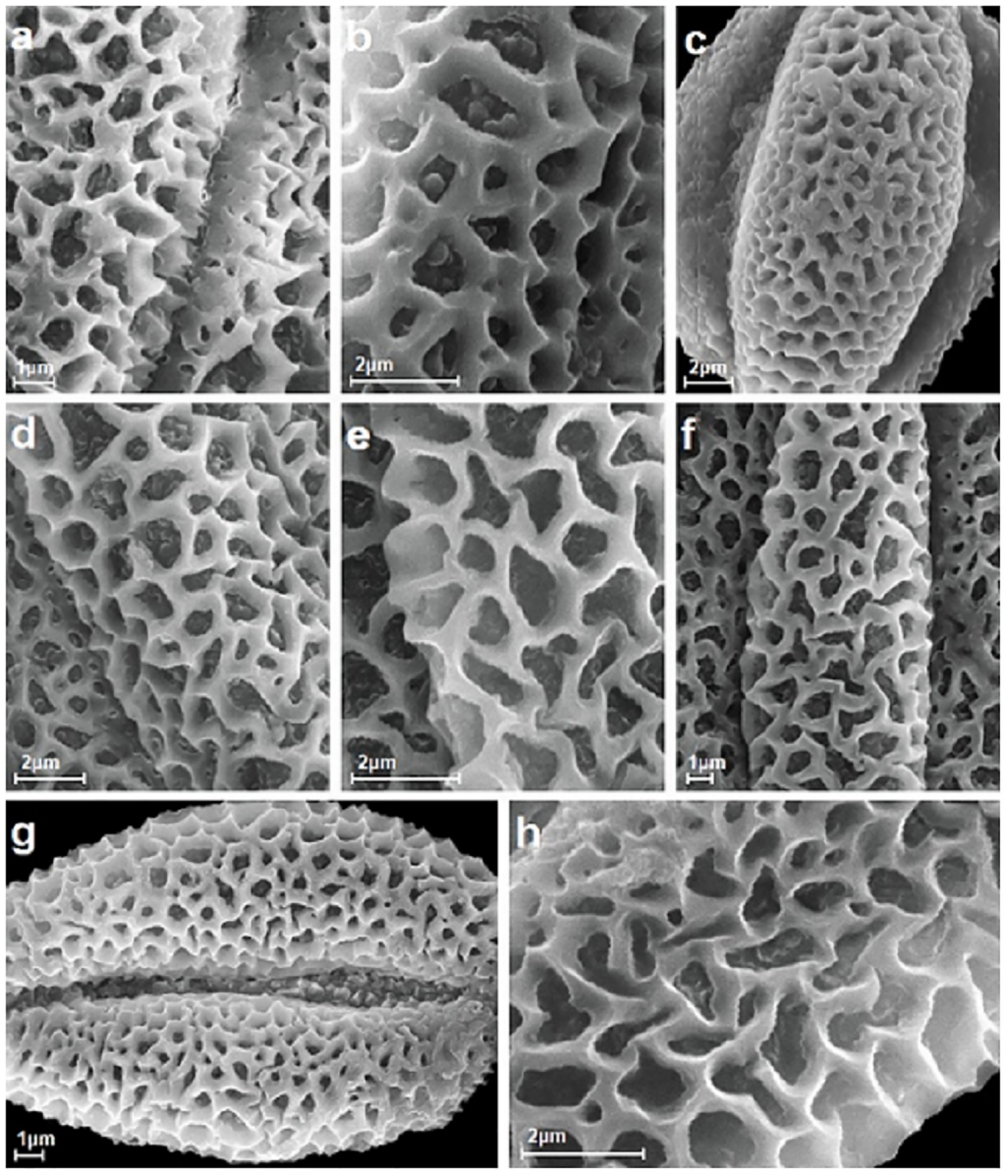
Reticulate exine ornamentation from types 1 and 2 with lumen, muri and free-standing columellae visible, A-H. A, *S*. *alba*; B, *S*. *aurita*; C, *S*. *caprea*; D, *S*. *cinerea*; E, S. *daphnoides*; F, *S*. *dasyclados*; G, *S*. *eleagnos*; H, *S*. *fragilis*.

**Fig 3:**
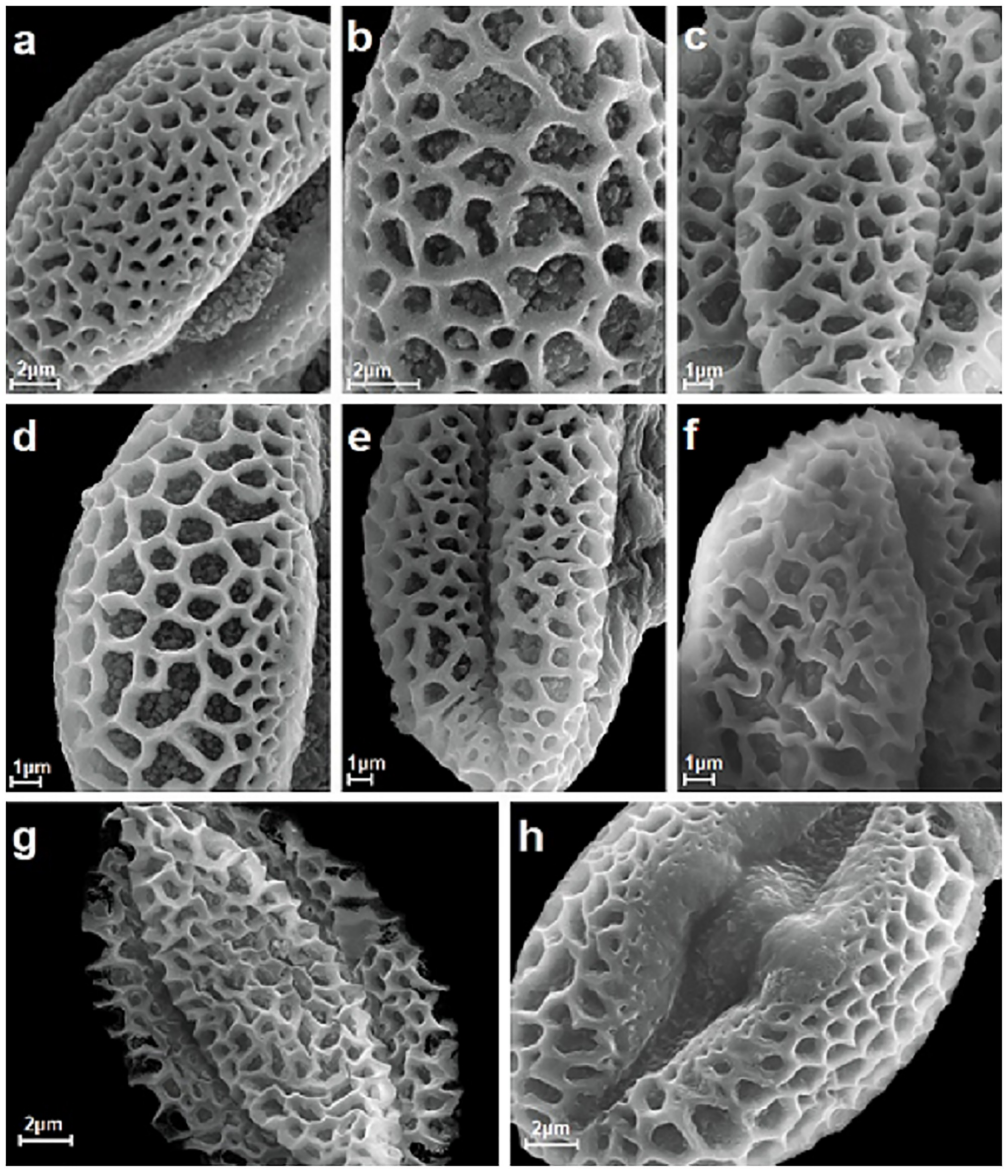
Reticulate exine ornamentation from types 1 and 2 with lumen, muri and free-standing columellae visible, A-H. A, *S*. *hastata*; B, *S*. *alpina*; C, *S*. *lapponum*; D, *S*. *myrsinifolia*; E, *S*. *myrtilloides*; F, *S*. *pentandra*; G, *S*. *phylicifolia*; H, *S*. *purpurea*.

**Fig 4:**
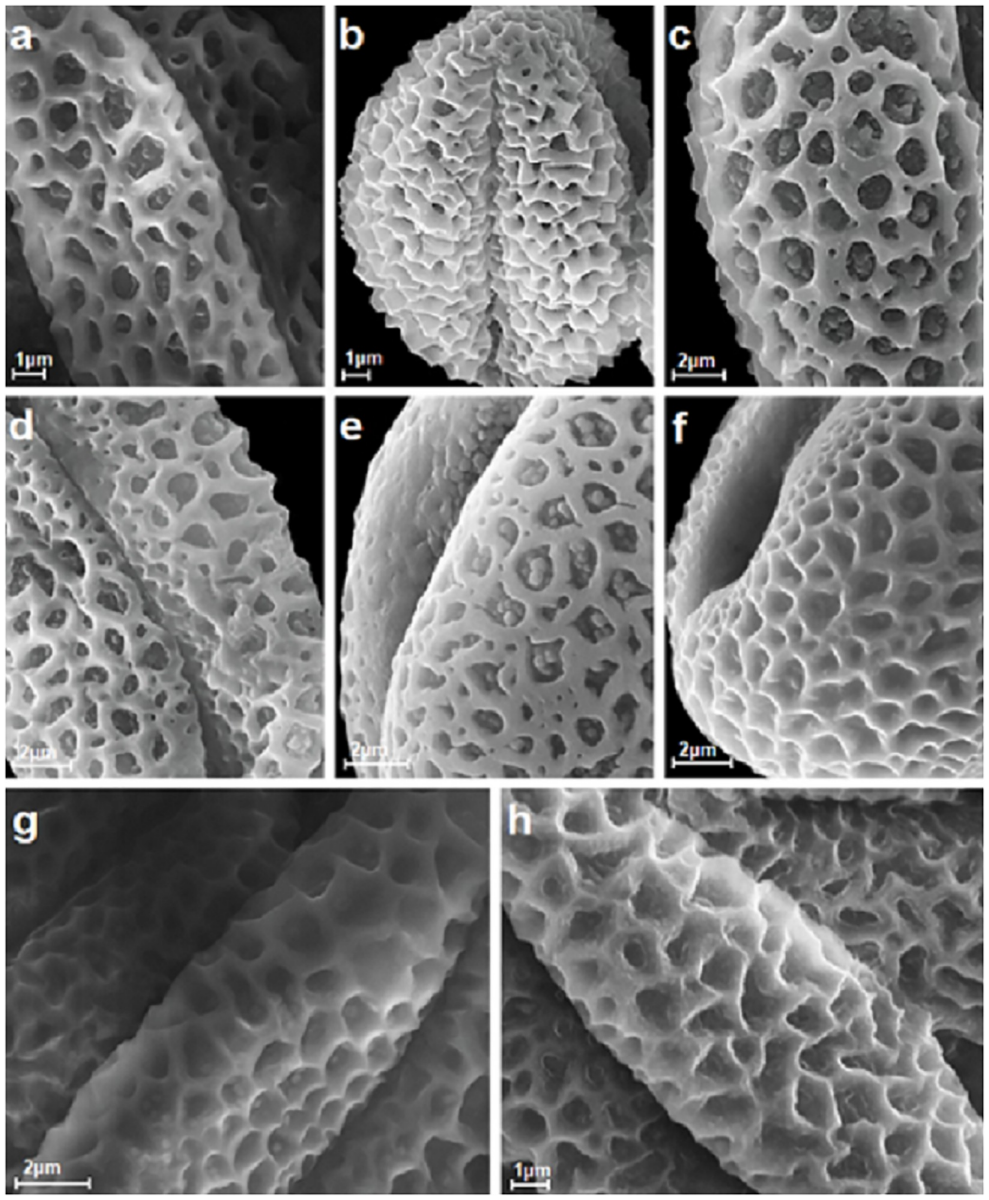
Reticulate exine ornamentation from types 1 and 2 with lumen, muri and free-standing columellae visible, A-H. A, *S*. *repens*; B, *S*. *reticulata*; C, *S*. *retusa*; D, *S*. *silesiaca*; E, *S*. *starkeana*; F, *S*. *triandra*; G, *S*. *rosmarinifolia*; H, *S*. *viminalis*.

A description of the pollen grain morphology of the *Salix* taxa samples under analysis is given below and illustrated in the SEM photographs (Figs 1A–1H to 4A–4H). The morphological observations for the quantitative features are summarized in Table 2.

**Table 2:** Minimal, mean and maximal values as well as coefficient of variation (cv, in %) for all observed traits and for particular *Salix* species.

Species	P			E			P/E			Le		
	mean	min-max	cv	mean	min-max	cv	mean	min-max	cv	mean	min-max	cv
*S*. *alba*	23.13	21.23–24.97	4.38	19.61	17.29–22.00	6.11	1.183	1.057–1.309	5.73	18.08	15.45–19.89	6.10
*S*.*alpina*	20.48	18.94–21.81	4.03	16.37	15.28–17.92	4.09	1.253	1.114–1.353	4.58	17.22	14.96–18.38	5.24
*S*. *aurita*	20.73	17.89–22.62	5.24	17.9	16.22–19.41	4.28	1.159	1.048–1.266	4.53	16.56	13.65–19.11	9.33
*S*. *caprea*	23.60	21.09–26.98	5.91	17.73	16.19–19.38	4.11	1.334	1.186–1.570	7.38	18.74	15.33–20.93	9.07
*S*. *cinerea*	24.93	22.19–26.42	3.72	20.99	19.09–23.26	4.65	1.190	1.046–1.328	5.38	19.86	16.31–22.55	7.20
*S*. *daphnoides*	22.69	20.22–25.85	6.86	18.45	16.92–19.96	4.96	1.230	1.088–1.342	5.27	18.56	16.18–21.26	8.08
*S*. *dasyclados*	24.91	22.20–28.11	5.87	20.55	18.20–22.72	5.23	1.214	1.056–1.364	5.67	20.83	17.33–24.05	7.88
*S*. *eleagnos*	23.43	20.81–25.65	5.48	19.85	18.21–21.71	4.42	1.181	1.043–1.368	5.70	19.36	15.52–22.86	8.79
*S*. *fragilis*	22.93	19.7–26.22	6.67	20.6	17.46–23.88	6.83	1.114	0.957–1.213	4.69	18.59	14.30–22.14	10.86
*S*. *hastata*	21.92	19.35–24.28	5.22	17.73	15.95–19.36	4.70	1.238	1.079–1.386	6.05	17.35	14.84–20.60	9.28
*S*. *lapponum*	25.33	22.30–27.31	5.83	19.94	18.32–22.26	5.29	1.271	1.090–1.392	4.82	20.77	16.51–23.55	8.05
*S*. *myrsinifolia*	24.02	20.42–27.15	7.28	20.31	17.55–22.18	6.16	1.184	1.029–1.354	6.30	20.41	16.22–23.60	8.42
*S*. *myrtilloides*	23.26	19.93–25.43	5.80	20.69	16.00–23.33	8.89	1.129	1.017–1.290	6.31	20.27	15.74–24.67	8.66
*S*. *pentandra*	18.46	17.42–19.9	3.53	17.14	15.56–18.72	4.29	1.078	0.966–1.155	4.20	14.62	12.93–16.07	6.21
*S*. *phylicifolia*	18.86	16.86–21.26	6.18	15.51	13.85–17.58	5.65	1.219	0.999–1.349	6.44	15.41	13.36–20.16	10.34
*S*. *purpurea*	20.70	18.60–22.04	4.39	17.12	15.96–19.02	4.38	1.211	1.054–1.354	5.96	16.69	14.65–19.06	7.03
*S*. *repens*	22.79	20.22–24.79	4.64	18.62	16.65–20.46	5.13	1.226	1.099–1.396	5.82	18.70	16.25–21.24	6.43
*S*. *reticulata*	17.34	15.75–19.27	5.30	15.62	13.58–17.82	5.83	1.113	0.997–1.281	5.71	13.66	11.54–15.59	8.11
*S*. *retusa*	26.94	24.02–28.71	5.07	24.91	21.12–27.4	6.38	1.084	0.942–1.195	5.49	21.89	17.93–25.78	8.85
*S*. *rosmarinifolia*	21.09	18.30–23.38	6.27	16.99	14.47–18.84	6.29	1.244	1.025–1.449	7.05	16.02	14.08–18.93	8.26
*S*. *silesiaca*	21.24	18.88–23.44	5.30	17.93	16.45–19.96	5.31	1.188	1.001–1.341	7.87	15.83	14.02–17.63	6.94
*S*. *starkeana*	25.24	22.74–27.62	4.43	21.09	19.12–23.02	4.99	1.199	1.083–1.305	4.92	20.77	18.01–23.65	7.30
*S*. *triandra*	18.83	16.70–20.91	6.07	15.82	14.19–17.82	5.40	1.193	1.011–1.307	6.49	15.03	12.64–16.87	7.59
*S*. *viminalis*	21.52	18.43–23.87	5.95	18.23	16.47–19.94	5.27	1.183	1.030–1.366	7.34	17.57	14.95–19.33	7.10
LSD_0.01_	1.049			0.895			0.06			1.255		
*F* statistic	117.74***			132.19***			21.26***			69.74***		
Species	Ex			Le/P			Ex/P			Ex/E		
	mean	min-max	cv	mean	min-max	cv	mean	min-max	cv	mean	min-max	cv
*S*. *alba*	1.746	1.45–2.14	10.27	0.782	0.719–0.849	4.76	0.076	0.0585–0.0892	9.55	0.089	0.070–0.111	10.32
*S*. *alpina*	1.59	1.39–1.76	6.51	0.842	0.754–0.935	5.71	0.078	0.0666–0.0887	7.95	0.097	0.083–0.112	6.53
*S*. *aurita*	1.536	1.29–1.83	10.14	0.799	0.695–0.888	7.08	0.074	0.0592–0.0853	9.79	0.086	0.070–0.099	9.97
*S*. *caprea*	1.437	1.25–1.65	6.42	0.794	0.710–0.881	6.77	0.061	0.0530–0.0738	8.85	0.081	0.071–0.092	6.41
*S*. *cinerea*	1.707	1.32–1.95	8.25	0.796	0.659–0.900	6.20	0.069	0.0500–0.0798	8.93	0.081	0.062–0.102	9.37
*S*. *daphnoides*	1.764	1.52–1.96	7.23	0.818	0.718–0.925	5.68	0.078	0.0673–0.0888	7.30	0.096	0.083–0.111	6.81
*S*. *dasyclados*	1.709	1.58–1.94	5.32	0.836	0.735–0.888	4.75	0.069	0.0596–0.0774	7.03	0.083	0.070–0.097	7.19
*S*. *eleagnos*	1.814	1.52–2.09	9.09	0.827	0.652–0.949	7.62	0.077	0.0662–0.0913	8.43	0.092	0.073–0.110	10.33
*S*. *fragilis*	1.413	1.22–1.88	11.44	0.810	0.659–0.905	7.08	0.062	0.0525–0.0839	11.95	0.069	0.058–0.094	13.15
*S*. *hastata*	1.766	1.59–1.99	6.27	0.792	0.665–0.903	7.39	0.081	0.0700–0.0932	7.82	0.100	0.089–0.118	8.16
*S*. *lapponum*	1.726	1.48–2.06	8.27	0.820	0.731–0.902	5.48	0.068	0.0544–0.0924	11.05	0.087	0.070–0.101	8.78
*S*. *myrsinifolia*	1.827	1.51–2.04	7.35	0.851	0.767–1.076	6.82	0.076	0.0635–0.0882	8.69	0.090	0.076–0.106	7.79
*S*. *myrtilloides*	1.642	1.44–1.84	7.56	0.872	0.735–0.970	6.34	0.071	0.0592–0.0891	9.93	0.080	0.063–0.112	11.82
*S*. *pentandra*	1.533	1.30–1.87	10.39	0.793	0.714–0.908	6.76	0.083	0.0716–0.1051	10.37	0.089	0.076–0.110	9.86
*S*. *phylicifolia*	1.621	1.38–1.83	7.21	0.817	0.720–0.948	6.89	0.086	0.0719–0.1008	7.71	0.105	0.089–0.120	7.33
*S*. *purpurea*	1.421	1.25–1.68	7.36	0.807	0.696–0.904	6.22	0.069	0.0591–0.0804	7.63	0.083	0.071–0.099	8.71
*S*. *repens*	1.561	1.32–1.93	9.06	0.822	0.717–0.958	6.29	0.069	0.0533–0.0955	11.29	0.084	0.072–0.105	9.36
*S*. *reticulata*	1.617	1.37–1.88	8.77	0.788	0.696–0.927	6.66	0.093	0.0796–0.1137	10.09	0.104	0.086–0.125	9.32
*S*. *retusa*	1.900	1.51–2.20	8.93	0.813	0.683–0.926	7.42	0.071	0.0566–0.0837	9.28	0.076	0.060–0.089	9.88
*S*. *rosmarinifolia*	1.541	1.29–1.93	11.75	0.760	0.651–0.837	5.81	0.074	0.0616–0.0981	15.46	0.091	0.072–0.120	13.57
*S*. *silesiaca*	1.548	1.36–1.96	10.03	0.746	0.649–0.856	5.69	0.073	0.0622–0.0916	11.01	0.086	0.070–0.110	10.09
*S*. *starkeana*	1.800	1.51–2.06	7.78	0.824	0.713–0.946	6.73	0.071	0.0576–0.0814	8.81	0.086	0.070–0.098	8.37
*S*. *triandra*	1.529	1.28–1.83	11.41	0.799	0.701–0.885	6.24	0.081	0.0665–0.1036	12.64	0.097	0.072–0.125	12.51
*S*. *viminalis*	1.491	1.17–1.83	11.88	0.818	0.707–0.899	6.90	0.070	0.0538–0.0867	13.90	0.082	0.063–0.098	11.07
LSD_0.01_	0.123			0.044			0.006			0.007		
*F* statistic	27.87***			8.27***			29.94***			30.95***		

P ˗ length of the polar axis, E ˗ equatorial diameter, Le ˗ length of the ectoaperture, Ex ˗ exine thickness, cv ˗ coefficient of variation (%).

The pollen grains of the studied *Salix* taxa were radially symmetrical, isopolar monads (Fig 1A–1E). They represented two pollen types: tricolpate (e.g. *S*. *alba*, *S*. *purpurea*, *S*. *reticulata*, and *S*. *retusa*) and tricolporate (e.g. *S*. *cinerea*, *S*. *daphnoides*, *S*. *fragilis*, *S*. *repens*, and *S*. *serpyllifolia*). Dicolpate pollen also appeared, but very rarely (e.g. *S*. *caprea*).

**Fig 5:**
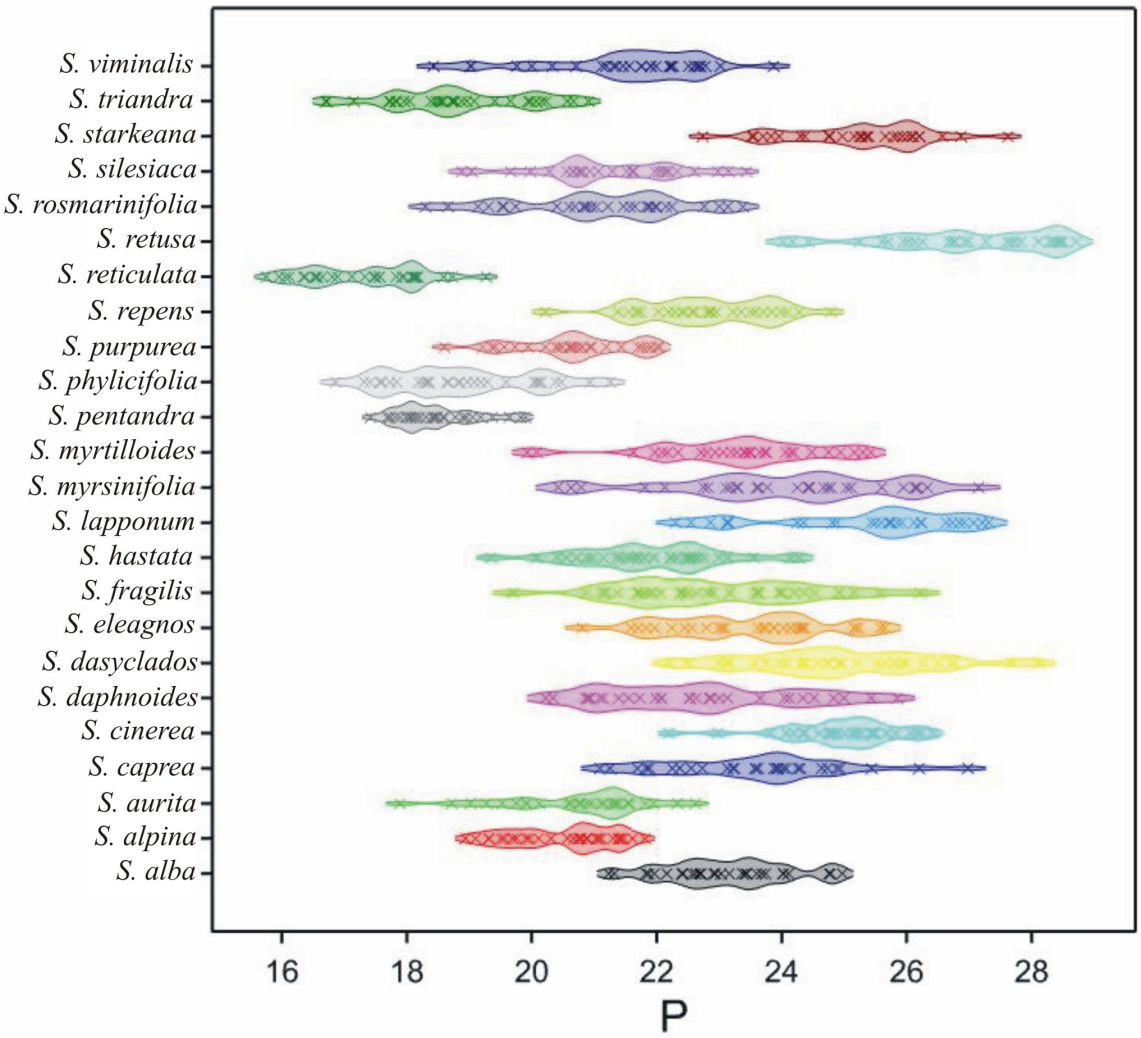
Density plot of P by *Salix* species. The range of violins mean minimal and maximal values for particular species. The points (crosses inside violins) are plotted along a line, with a kernel density smooth on either side to indicate the density of points along the line. Different colours are for different species.

The studied pollen grains, according to Erdtman’s [15] spore size classification, were both small- and medium-sized, with the longest axis (P) ranging between15.7 and28.7 μm (Table 2, Fig 5). Generally, the small-sized class of pollen prevailed in 20 *Salix* species, including 13 species (*S*. *alba*, *S*. *aurita*, *S*. *hastata*, *S*. *alpina*, *S*. *pentandra*, *S*. *phylicifolia*, *S*. *purpurea*, *S*. *repens*, *S*. *reticulata*, *S*. *rosmarinifolia*, *S*. *silesiaca*, *S*. *triandra*, and *S*. *viminalis*) with only small-sized pollen grains. In another five species (*S*. *caprea*, *S*. *daphnoides*, *S*. *eleagnos*, *S*. *fragilis*, and *S*. *myrtilloides*), the participation of medium-sized pollen grains (with P axis over 25 μm in length) did not exceed 20%, in two species (*S*. *dasyclados* and *S*. *myrsinifolia*), the percentage of class grains of such a size totalled ca 30–40%, and in one species (*S*. *cinerea*), the number of small and medium-sized grains was the same. The predominance of medium-sized grains (more than 70%) was observed in only two species (*S*. *lapponum* and *S*. *retusa*).

Totally, the average length of the polar axis (P) was 22.27 μm, with extreme values of 15.75 μm in *S*. *reticulata* and 28.71 μm in *S*. *retusa* (Table 2, Fig 5). At the same time, these two species were characterised by, on average, the smallest P (in *S*. *reticulata*– 17.34 μm) and the largest (in *S*. *retusa–* 26.94 μm). On average, relatively small P axes were also observed in *S*. *pentandra*, *S*. *phylicifolia*, and *S*. *triandra* (less than 19.00 μm) and relatively high values of P axes (ca 25 μm) were noticed in *S*. *cinerea*, *S*. *dasyclados*, *S*. *lapponum* and *S*. *starkeana*.

Considering all the studied species, the mean length of the equatorial diameter (E) was 18.94 μm, the smallest value of this feature totaling 13.58 μm (in *S*. *reticulata*) and the largest amounting to 27.40 μm (in *S*. *retusa*) (Table 2). The shortest mean equatorial diameter occurred in *S*. *phylicifolia* (15.51 μm), while the longest was in *S*. *retusa* (20.40 μm). On average, relatively small E axes (less than 16.00 μm) were also observed in *S*. *reticulata* and *S*. *triandra* and relatively high values of E axes (above 20 μm) in *S*. *cinerea*, *S*.*dasyclados*, *S*.*fragilis*, *S*. *myrsinifolia*, *S*. *myrtilloides* and *S*. *starkeana*.

The outline in polar view was mostly trilobate, less frequently circular or elliptic, whereas in equatorial view the outline was mostly elliptic and only sporadically circular (Fig 1A–1E).

**Fig 6:**
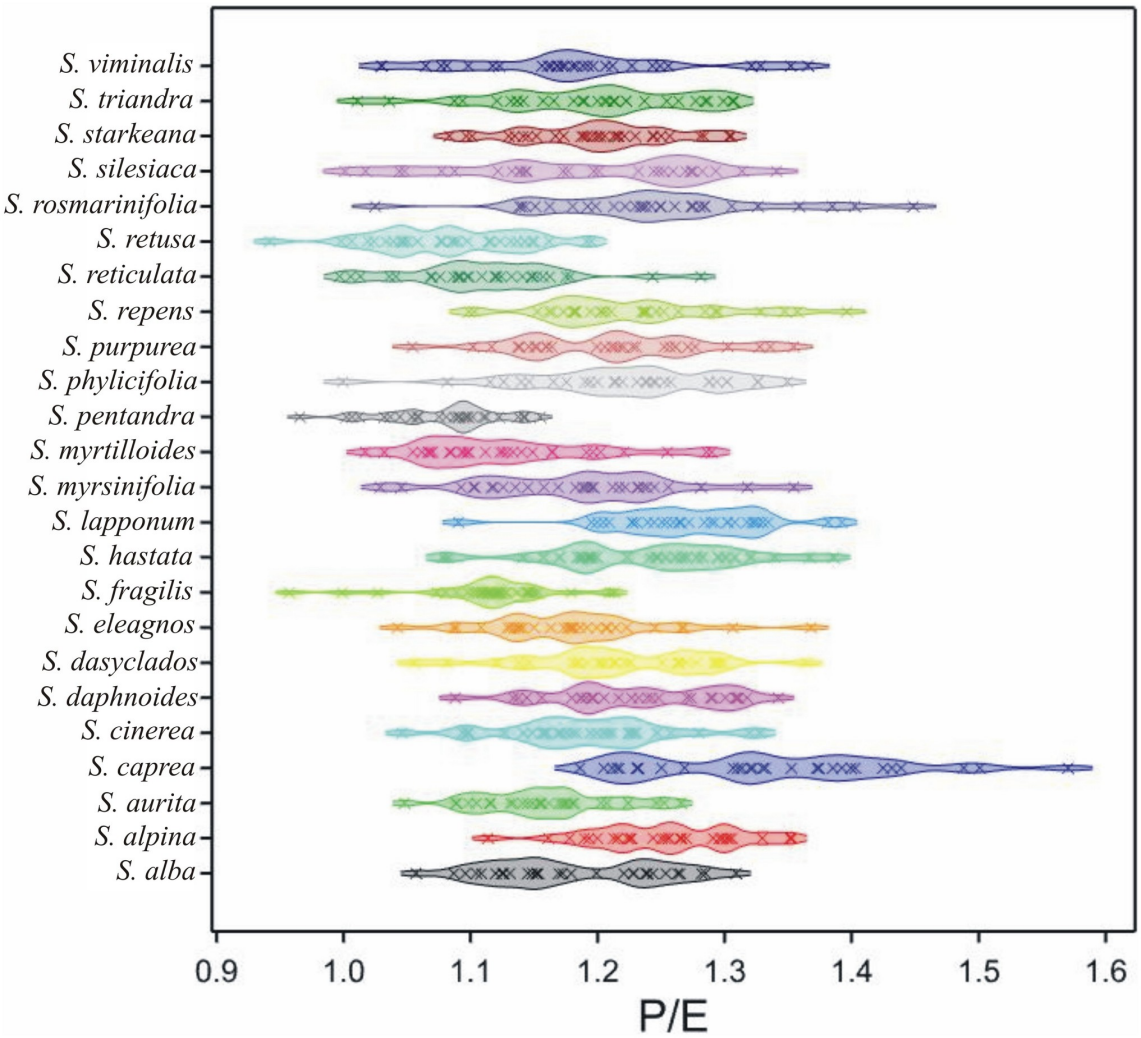
Density plot of P/E by *Salix* species. The range of violins mean minimal and maximal values for particular species. The points (crosses inside violins) are plotted along a line, with a kernel density smooth on either side to indicate the density of points along the line. Different colours are for different species.

Generally, the mean P/E ratio was 1.19, and ranged from 0.82 in *S*. *retusa* to 1.57 in S. *caprea* (Table 2). On average, the smallest value of P/E ratio was in *S*. *pentandra* and *S*. *retusa* (1.08) and the largest in *S*. *caprea* (1.33) The largest range of P/E ratio was found in S. *caprea* and the smallest one in *S*. *pentandra* (Table 2, Fig 6).

In all the investigated *Salix* species, subprolate (62.7%) and prolate spheroidal (30.8%) types of pollen shape classes predominated. Sporadically, prolate types of pollen shapes (approx. 5%) were noted, whereas the total participation of oblate-spheroidal and spheroidal pollen classes did not exceed 1%. The most frequently prolate-spheroidal class was visible in *S*. *pentandra* (87%), then in *S*. *fragilis*, *S*. *myrtilloides* and *S*. *reticulata* (67–80%). The subprolate class of pollen shape was the most characteristic in *S*. *alpina* and *S*. *lapponum* (90%), while a large participation of this class was also found in *S*. *cinerea*, *S*. *daphnoides*, *S*. *dasyclados*, *S*. *hastata*, *S*. *phylicifolia*, *S*. *repens* and *S*. *starkeana* (80–87%).

The exine consisted of two layers: the sexine was usually slightly thicker than the nexine. Totally, the mean exine thickness was 1.63 μm (with a range of 1.17 μm in *S*. *viminalis* up to 2.20 μm in *S*. *retusa*) (Table 2). On average, the exine was the thinnest in *S*. *fragilis* (1.41 μm) and *S*. *purpurea* (1.42 μm), while the thickest occurred in *S*. *retusa* (1.90 μm).

Totally, the relative thickness of the exine (Ex/P ratio) averaged 0.07 (ranging from 0.05 to 0.11) and the Ex/E ratio was 0.09 (0.06–0.13) (Table 2). The above results indicated that the exine was characterised by an almost identical thickness along the entire pollen grain.

The pollen grains under analysis usually had three apertures ˗ colpori or colpi. The ectoapertures were arranged meridionally, regularly, and they were quite evenly spaced and long: with a mean length of 18.03 (11.54–24.67) μm (Table 2). On average, the length of the ectoapertures (colpi) constituted 81% (from 65 to 97%) of the polar axis length, with the shortest colpi found in *S*. *reticulata* (11.54 μm) and the longest in *S*. *myrtilloides* (24.67 μm). The colpi were wide and elliptic in outline. The ectoaperture membrane was usually ornamented (granulate or microgranulate) and sometimes partly ornamented and partly psilate (Fig 1F–1H). In the ectoapertures of the majority of the studied species, margo was observed (e.g. in *S*. *alba*, *S*. *capraea*, *S*. *cinerea*, *S*. *daphnoides*, *S*. *repens*, *S*. *reticulata*, *S*. *retusa*, *S*. *serpyllifolia*, and *S*. *triandra*). The margines were quite wide, darker than the rest of the ectoaperture, psilate from the ectoaperture side and reticulate with a few, diffused lumina with small, but different diameters from the mesocolpium side.

Exine ornamentation was reticulate, and created by the lumina and muri, which varied in shape and size (Figs 1–4). The studied pollen grains were heterobrochate, which means that the pollen had a reticulate pollen wall with lumina of different sizes and often irregular outlines. Their diameters ranged from 0.4 to 2.0 μm. The lumina were at its maximum size in the center of the mesocolpium area and then gradually or suddenly decreased in size towards the poles and colpi. In many studied species within the lumina, single to numerous free-standing columellae of different heights were present (e.g. in *S*. *cinerea*, *S*. *daphnoides*, *S*. *repens*, and *S*. *retusa*) (Figs 2D, 2E, 4A and 4C). These columellae were single or did not occur in, for example, *S*. *alba*, *S*. *caprea*, *S*. *purpurea*, or *S*. *reticulata*) (Figs 2A, 2C, 3H and 4B). The columella shape varied from spheroid to elliptical and polygonal, with rounded or triangular angles. The features of the muri were also very variable. The differences were in the height, width, and the rounded or angled margins of the muri. The borders of the muri were undulate or erect (see: Pollen key).

The investigated pollen of the individual *Salix* species was classified, based on the exine ornamentation classification proposed by Sohma [28], into two types (1 and 2). In that study, eight exine ornamentation types were distinguished. The greatest number of the studied species (17) belonged to type 2, which was characterized by wedge-shaped muri, considerably variable in width. The differences in the shape and the dimentions of the lumina were also considerable. Type 1 was represented by three species (*S*. *alba*, *S*. *eleagnos*, and *S*. *reticulata*; Figs 2A, 2G and 4B). According to Sohma [28], type 1 was very specific and consisted of pollen grains with conspicuous keeled muri. Indeed, the muri were often acutely pointed at the trifurcate points where the immediate neighboured meshes joined together. The side walls of the muri delimiting the meshes were relatively straight, curved, or sinuous. The lumina were almost isodiametric and ellipsoidal to round polygonal in outline, and separated by relatively narrow muri. Among the examined species, four had both types of exine ornamentation (*S*. *phylicifolia*, *S*. *romarinifolia*, *S*. *triandra*, and *S*. *viminalis*; Figs 3G, 4G, 4F and 4H).

### Pollen key

1. Edges of muri clearly acutely pointed ……………………………………….…………..…2

1.* Edges of muri obtuse, at most faintly wedged……………………………………………..5

2. Lumina similar in outline, varied in size, but less than 1 μm in diameter….………….…….3

2.* Lumina varied both in size and outline, at least part of the lumina larger than 1 μm in the longest diameter…………..…………………………………..…….……………………..….4

3. All long muri prominently wedged……………………..…………………..….. *S*. *reticulata*

3.* Muri especially keeled at the junction of 3–4 adjacent lumina…….…………….*S*. *eleagnos*

4. The most often lumina irregularly rounded polygonal in outline. Only sporadically incomplete fusion of muri……………..…….….……….*S*. *pentandra*, *S*. *alba*, *S*. *daphnoides*

4.* Numerous lumina elongated in outline due to sinuosity of the muri. Incomplete fusion of muri often visible…………..………..…*S*. *fragilis*, *S*. *phylicifolia*, *S*. *viminalis*, *S*. *dasyclados*

5. Muri of variable width, but relatively wide and and with oblate edges….………….………6

5.* Muri of similar width, with non-flattened edges………………….……..……..…..………9

6. Lumina mixed with numerous faint perforations……………………………….…..……….7

6*. Perforations only sporadically visible………..………………………….….….…..*S*. *repens*

7. Within lumina numerous free columellae, often of the same length as muri……*S*. *starkeana*

7.* Within lumina free columellae lower than muri………………………….….…………….8

8. The longest diameter of lumina rather smaller than 2 μm………………………….*S*. *hastata*

8.* At least part of the lumina larger than 2 μm in the longest diameter…….………..*S*. *retusa*

9. Lumina relatively large in diameter, sometimes above 2 μm.…..*S*. *myrsinifolia*, *S*. *alpina*

9.* Lumina up to 2 μm in diameter…………………………….……..…..……..……………10

10. Lumina ± similar in outline…………….…..…………………………..………..………..11

10.* Lumina varied in outline, with the participation of elongated….….……………………12

11. Colpus margo distinct…………………………………………..………..………*S*. *triandra*

11.* Colpus margo obscure………………………*S*. *myrtilloides*, *S silesiaca*, *S*. *rosmarinifolia*

12. Colpus membrane granular…………………………..………………..…………..*S*. *caprea*

12.* Colpus membrane without granula…….…*S*. *cinerea*, *S*. *aurita*, *S*. *lapponum*, *S*. *purpurea*

### Pollen grain variability

The results of the MANOVA indicated that all the samples were significantly different with regard to all of the eight quantitative traits (Wilk’s λ = 0.01053; F_23;696_ = 23.44; and *P*<0.0001). The results of ANOVA indicated that the main effects of the species were significant for all eight observed traits (Table 2). The mean values, ranges and coefficients of variation (cv) for the observed traits indicated a high variability among the tested samples and significant differences were found in terms of all the analysed morphological traits (Table 2).

The intraspecific and inter-individual variability of the *Salix* pollen grains were studied based on eight selected quantitative features. Statistical analysis for the studied traits indicated a high variability among the tested species. The most variable biometric traits were P, E and Le, while lower variability occurred in P/E, Le/P and d/E (Table 2).

**Fig 7:**
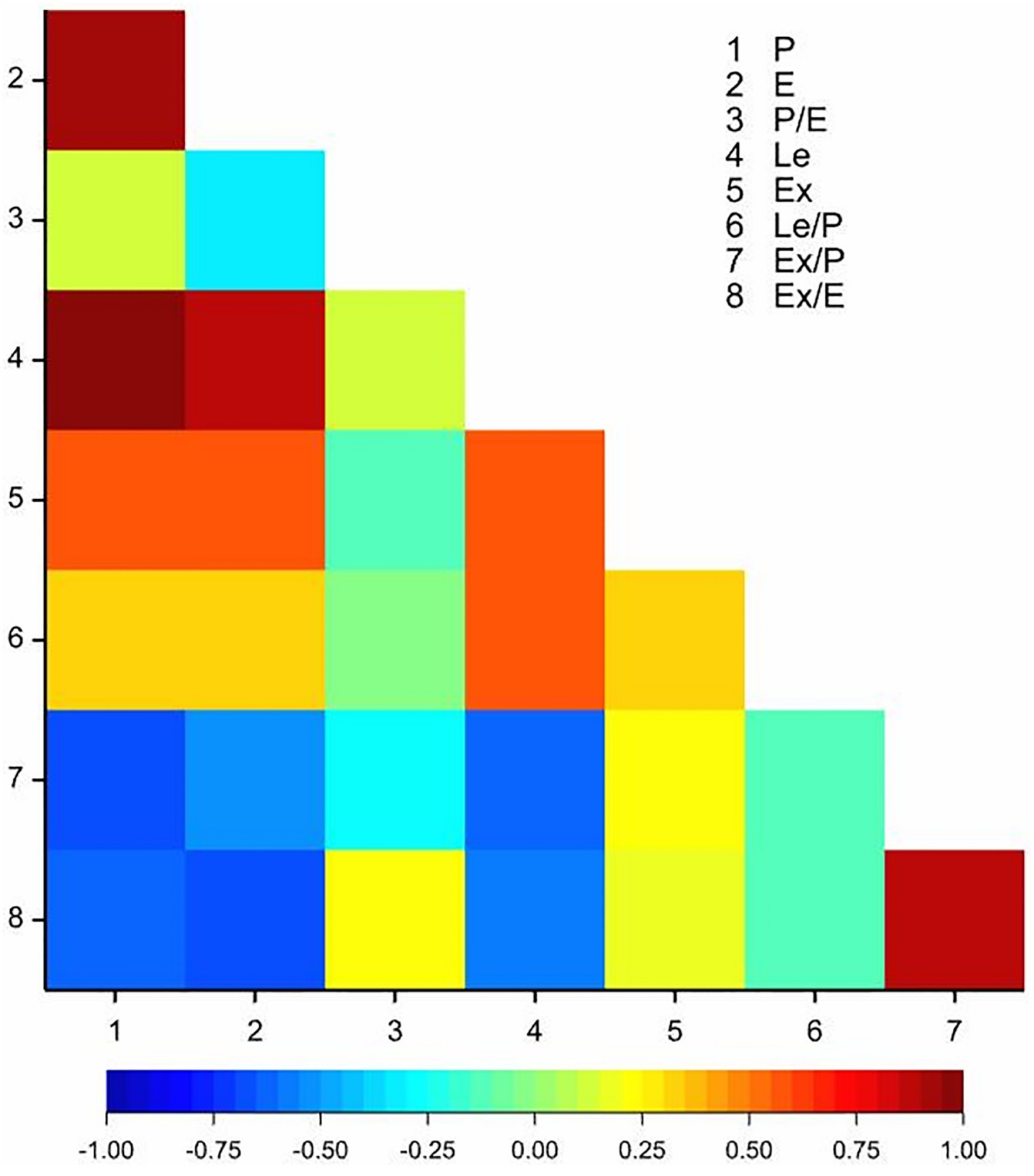
Heatmap for linear Pearson’s correlation coefficients between eight morphological traits of studied *Salix* species. P–the length of the polar axis, E–equatorial diameter, Le–the length of the ectoaperture, Ex–the exine thickness, P/E–the length of the polar axis and equatorial diameter ratio, Le/P–the length of the ectoaperture and the length of the polar axis ratio, Ex/P–the exine thickness and the length of the polar axis ratio, Ex/E–the exine thickness and equatorial diameter ratio (*r*_0.05_ = 0.40); colours mean level of correlation.

The correlation analysis performed indicated statistically significant correlation coefficients for 14 out of 28 coefficients (Fig 7). P was positively correlated with E (*r* = 0.90), Le (0.97), Ex (0.56), and negatively with Ex/P (-0.68) and Ex/E (-0.61). Trait E was correlated with: Le (0.88), Ex (0.58), Ex/P (-0.54) and Ex/E (-0.68) (Fig 3). Trait Le correlated with: Ex (0.58), Le/P (0.56), Ex/P (-0.63) and (-0.57). Additionally, Ex/P was correlated with Ex/E (0.88, Fig 7).

**Fig 8:**
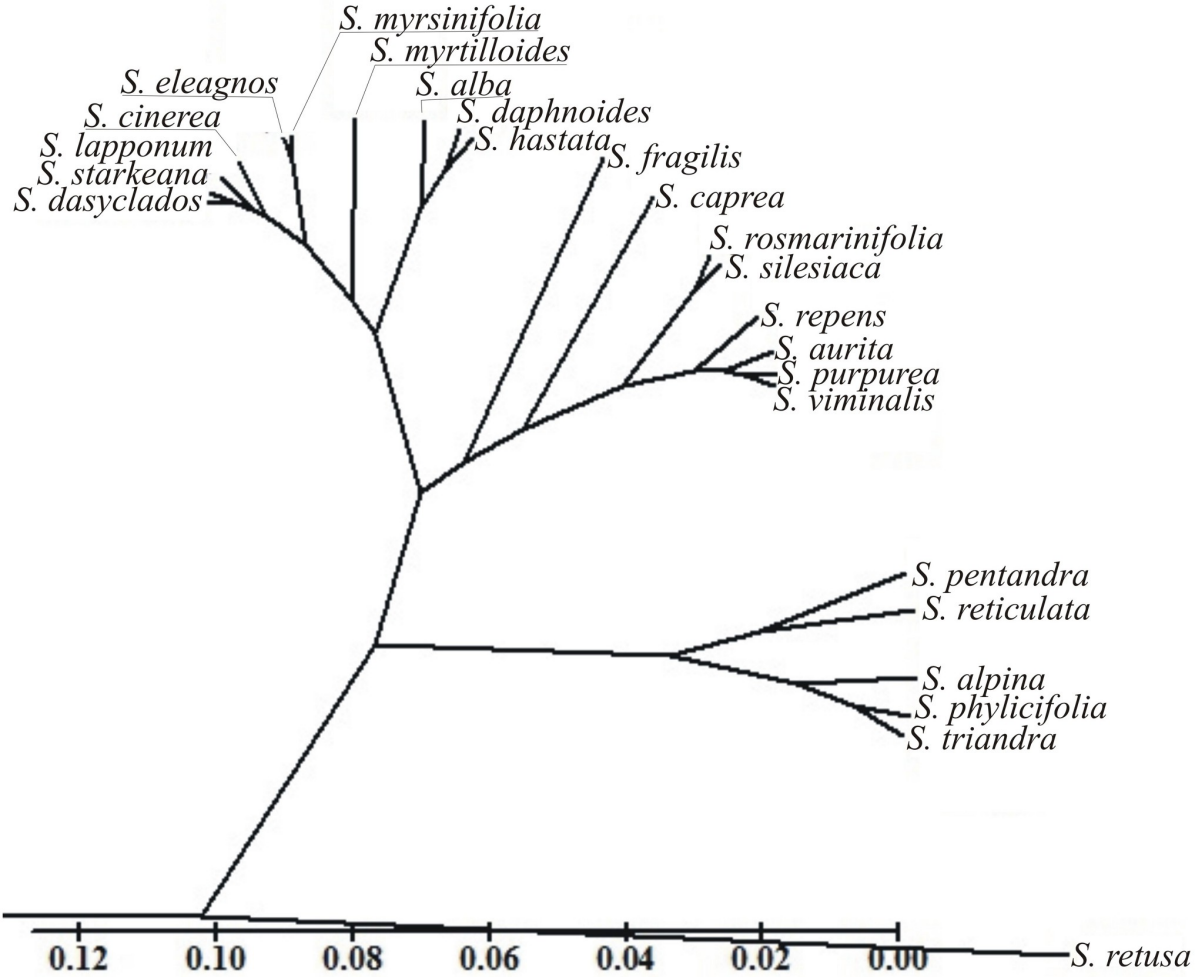
Dendrogram of cluster groupings of *Salix* species based on all eight morphological traits: The length of the polar axis, equatorial diameter, the length of the ectoaperture, the exine thickness, the length of the polar axis and equatorial diameter ratio, the length of the ectoaperture and the length of the polar axis ratio, the exine thickness and the length of the polar axis ratio and the exine thickness and equatorial diameter ratio.

In the presented dendrogram, as a result of agglomeration grouping using the Euclidean distance method, all the examined *Salix* species were divided into four groups (Fig 8). The first group (I) comprised one species*—S*. *retusa*, while the second one (II) consisted of five species. The third group (III) included eight species and the final one (IV) comprised ten species. The most distinct species was *S*. *retusa* from group I, while from the other groups, *S*. *caprea* and *S*. *fragilis* (group III) and *S*. *myrtilloides* (group IV) were also distinct.

Based on the dendrogram, taxonomic relationships between the studied species were analysed at the subgenus and section level. They belonged to two subgenera (*Salix* and *Chamaetia*/*Vetrix*) and formed four groups (Table 2, Fig 8). In general, willows did not form groups consistent with the currently used taxonomic division of the genus *Salix* into subgenera and sections. They fell into four clades with different number of species, including a separate group with only one species *S*. *retusa*. Species from the subgenus *Salix* belonged to three different groups. Similarly representatives of most numerous sections, as *Chamaetia* and *Vetrix*, were scattered.

Multi-traits distances between the studied species determined by Mahalanobis distances showed that the most similar were *S*. *eleagnos* and *S*. *myrsinifolia* (0.81), while *S*. *viminalis*—*S*. *aurita* and *S*. *lapponum*—*S*. *dasyclados* were also similar (1.08) (Table 3). The most distinct species were *S*. *retusa* and three willows—*S*. *reticulata* (11.20), *S*. *phylicifolia* (9.86) and *S*. *pentandra* (9.57) (Table 3).

**Table 3:** Mahalanobis distances between studied species of *Salix*.

Species	*S*. *alba*	*S*. *aurita*	*S*. *caprea*	*S*. *cinerea*	*S*. *daphnoides*	*S*. *dasyclados*	*S*. *eleagnos*	*S*. *fragilis*	*S*. *hastata*	*S*. *alpina*	*S*. *lapponum*	*S*. *myrsinifolia*	*S*. *myrtilloides*	*S*. *pentandra*	*S*. *phylicifolia*	*S*. *purpurea*	*S*. *repens*	*S*. *reticulata*	*S*. *retusa*	*S*. *rosmarinifolia*	*S*. *silesiaca*	*S*. *starkeana*	*S*. *triandra*	*S*. *viminalis*
*S*. *alba*	0																							
*S*. *aurita*	2.52	0																						
*S*. *caprea*	3.90	3.85	0																					
*S*. *cinerea*	1.94	3.93	4.31	0																				
*S*. *daphnoides*	1.49	2.31	3.87	2.85	0																			
*S*. *dasyclados*	2.23	3.83	4.17	1.31	2.57	0																		
*S*. *eleagnos*	1.14	3.03	4.37	2.06	1.41	1.85	0																	
*S*. *fragilis*	3.16	3.35	4.80	2.88	3.82	3.28	3.60	0																
*S*. *hastata*	2.11	2.18	4.04	3.63	1.16	3.50	2.38	4.11	0															
*S*. *alpina*	3.78	2.60	4.17	5.09	2.81	4.90	4.03	4.71	2.16	0														
*S*. *lapponum*	2.52	4.06	3.78	1.79	2.62	1.08	2.27	4.00	3.53	4.93	0													
*S*. *myrsinifolia*	1.79	3.60	4.48	2.01	1.99	1.48	0.81	3.82	3.00	4.53	2.00	0												
*S*. *myrtilloides*	2.35	3.34	4.48	2.29	2.76	2.16	2.11	2.37	3.39	4.28	3.06	2.03	0											
*S*. *pentandra*	4.46	2.44	5.08	5.75	4.24	5.63	4.91	5.01	3.89	3.73	5.79	5.38	5.08	0										
*S*. *phylicifolia*	5.29	3.82	5.66	6.51	4.43	6.37	5.61	5.81	3.52	2.26	6.41	6.11	5.72	3.72	0									
*S*. *purpurea*	3.23	1.23	3.39	4.54	2.87	4.40	3.75	3.76	2.65	2.28	4.52	4.27	3.88	2.93	3.77	0								
*S*. *repens*	1.77	1.87	2.75	2.62	1.61	2.40	2.12	2.98	2.16	2.94	2.49	2.46	2.44	3.92	4.65	2.10	0							
*S*. *reticulata*	6.69	5.05	6.72	7.71	6.15	7.52	7.05	7.09	5.52	4.98	7.51	7.43	7.15	3.24	3.47	5.34	6.09	0						
*S*. *retusa*	6.16	8.09	7.95	5.18	7.12	5.82	6.12	5.99	7.56	8.72	6.43	5.86	5.27	9.57	9.86	8.59	7.07	11.20	0					
*S*. *rosmarinifolia*	3.07	1.76	3.14	4.47	2.74	4.43	3.75	4.10	2.11	2.22	4.42	4.29	4.10	3.07	3.32	1.52	2.34	4.92	8.30	0				
*S*. *silesiaca*	2.45	1.23	3.44	3.89	2.62	3.97	3.26	3.52	2.31	3.06	4.10	3.85	3.68	2.85	4.16	1.64	2.10	5.28	7.87	1.25	0			
*S*. *starkeana*	2.18	4.26	4.67	1.12	2.80	0.95	1.73	3.64	3.74	5.33	1.53	1.33	2.39	6.08	6.83	4.91	2.93	8.04	5.29	4.87	4.33	0		
*S*. *triandra*	4.68	2.70	4.95	6.03	3.97	5.88	5.11	5.18	3.16	2.09	5.94	5.65	5.27	2.47	1.52	2.61	3.91	3.28	9.75	2.37	3.05	6.38	0	
*S*. *viminalis*	2.30	1.08	3.08	3.44	2.22	3.37	2.81	3.01	2.40	2.66	3.56	3.26	2.88	3.09	4.25	1.25	1.14	5.59	7.62	1.96	1.60	3.84	3.27	0
D_critical_	**6.67**																							

**Fig 9:**
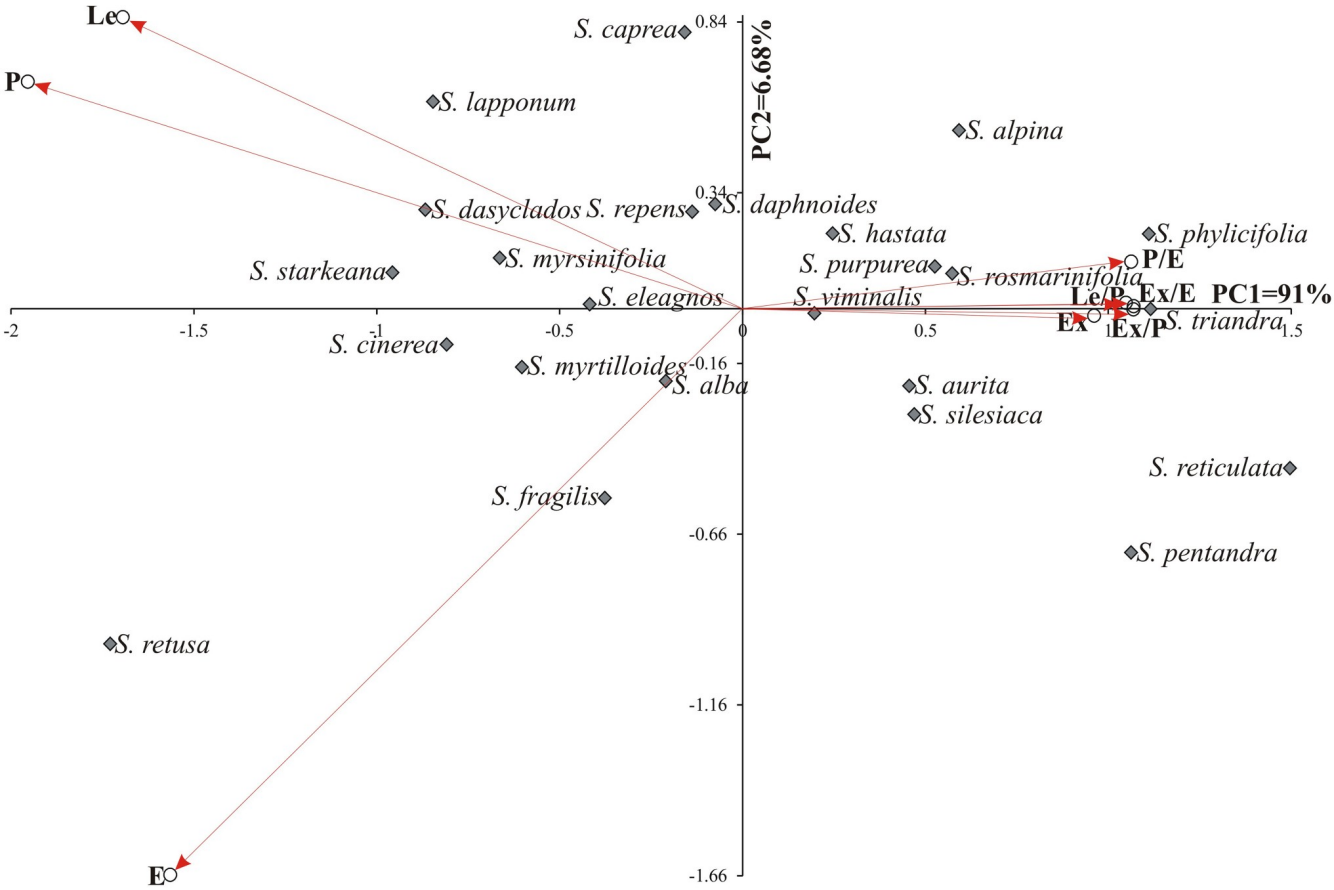
Biplot graph for studied species and observed traits of *Salix*.

Fig 9 shows the biplot of the variability of the pollen grain traits of 24 studied *Salix* species in terms of the first two principal components. In the graph, the coordinates of the point for particular species are the values for the first and second principal components, respectively. The first two principal components accounted for 97.68% of the total multivariate variability between the individual species. The goal of the study was to establish whether pollen grains collected from various *Salix* species growing in different habitat conditions (soil and climate) would differ from one another. Four groups of species were distinguished. The majority of the examined species were found in the first, large group (I). Just one or two willows (II–*S*. *retusa*, III–*S*. *reticulata*, *S*. *pentandra* and IV–*S*. *caprea* and *S*. *lapponum*) fell into the other three groups (Fig 9). The first group of species (I) was positively correlated with Ex, P/E, Le/P, Ex/E and Ex/P. Two species: *S*. *retusa* and *S*. *fragilis* were positively correlated with E (Fig 9).

## Discussion

Based on the palynological literature, it can be concluded that genus *Salix* has relatively uniform pollen grains, as pollen shape and size are roughly similar and exine ornamentation is reticulate [21, 22, 24, 25, 27, 28]. The results of this study confirmed all these conclusions. However, many researchers proved that a detailed analysis of exine sculpture can be used to distinguish the particular species [16, 19, 22, 25, 27–31].

The most precise ornamentation division in *Salix* was developed by Sohma [28]. Straka [16] concluded that the pollen grains of 30 European *Salix* species could be classified into six types, based mainly on the exine ornamentation and the character of the ectocolpus margin. He also noted that *S*. *silesiaca*, *S*. *herbacea*, *S*. *daphoides*, *S*. *pentandra* and *S*. *alba* were likely to be distinguished from each other, whereas most of the other taxa could not be distinguished. Based on pollen morphology, and mainly on exine ornamentation traits, Faegri and Iversen [19] identified four pollen types (*S*. *herbacea*, *S*. *polaris*, *S*. *pentandra* and *S*. *glauca*) in *Salix* species.

Several palynologists have used pollen traits for taxonomic studies on the genus *Salix*. Kim and Zsuffa [27] studied the pollen morphology of 15 *Salix* species belonging to six sections of the subgenus *Salix* and stated that, from a pollen-morphology point of view, the subgenus *Salix* was stenopalynous. The cited authors also examined the taxonomic relations and determined that *S*. *jessoensis* (section *Subalbae*) was the most distinct of the species studied. The species of the section *Humboldtianae* were most evolved in this subgenus, with a closer relationship to the section *Amygdalinae* than any other section of this subgenus. Sohma [28] examined the pollen grains of 72 taxa of the genus *Salix* and noted certain differences in the exine patterns. Based on these differences he described eight major types of reticulate exine ornamentation. In the study, height, width, the course of the muri and the diameter of the lumina were considered. Some of Soma’ s types were related to the genus *Salix* sections. Some sections were heterogeneous ˗ *Humboltdianae* (type 2), *Amygdalinae* (type 2), *Pentandrae* (type 1), *Salix* (type 6) and *Helix* (type 2a) had distinct types, other sections *Subalbae* (types 3, 5 and 8), *Longifoliae* (types 3 and 4), *Hastatae* (types 2–4, and 7), *Vetrix* (types 2 and 7) and *Daphnella* (types 1 and 2), while sections *Glabrella*, *Vimen* and *Subviminalis* were homogeneous (types 2). Babayi et al. [25] distinguished six pollen types (*S*. *alba*, *S*. *issatissensis*, *S*. *elbursensis*, *S*. *excelsa* and a type with three species ˗ *S*. *acmophylla*, *S*. *zygostemon* and *S*. *cinerea*). According to Sohma [28], section *Salix* conformed in its reticulation pattern with type 6, but the results from the study of Babayi et al. [25] did not support this. The species showed heterogeneous patterns, e.g. *S*. *alba* had type 6, *S*. *excelsa* ˗ types 3 and 6, and *S*. *issatissensis* ˗ type 1. Babayi et al. [25] reported that the exine was reticulate and the characteristics of the muri, such as the shape and size of the lumen, varied in different species. The lumen was isodiametric or heteromorphic and the patterns were orbicular, elliptic or polygonal with rounded angles. According to Babayi et al. [25], the exine features were different among the *Salix* species and could be used as diagnostic characteristics. In the current examination, the *Salix* species were divided into two pollen exine ornamentation types, based on the Sohma [28] classification. Within the studied species from the subgenera *Salix* and *Chamaetia/Vetrix*, two types of exine ornamentation (1 and 2) were found. This partly confirmed the thesis of Kim and Zsuffa [27], who claimed that, for example, the subgenus *Salix* had pollen of a similar structure. In contrast, Sohma [28] and Babayi et al. [25], who studied more species from these subgenera, distinguished five exine ornamentation types (1, 2, 4, 6, 7). According to the present research, the species from individual sections also usually had a similar exine ornamentation type. Only a detailed study of the exine ornamentation, contained in the attached pollen key, made it possible to distinguish eight of the 24 willow species studied, while the other species formed small groups of two to four species with very similar pollen characteristics.

The pollen grains of *Salix* taxa were described as tricolpate [e.g. 19, 25] or tricolporate [e.g. 21], or they were classified into both pollen classes [e.g. 28–31], just as in the presented studies.

Most of the authors cited above described the pollen grains of genus *Salix* as small (10–25 μm) [15, 24, 25, 28–31], and rarely as medium (25.1–50 μm) [15, 24, 28]. The researchers agreed that pollen size was not a useful feature to distinguish the individual *Salix* species. Only according to Babayi et al. [25] was the size of the pollen grains of particular *Salix* species very variable. The measurements made in this study yielded different results, confirmed by other authors. The range of the length of the polar axis (P) was narrow (15.75–28.71 μm). Moreover, most of the tested pollen grains were small, while others usually only exceeded 25.1 μm slightly, which was the maximum value for medium-sized grains. In addition, many species had similar ranges of the trait P (Fig 5), therefore pollen size is considered a poor criterion to distinguish the individual *Salix* species.

According to other authors, the pollen shape was various, oblate to subprolate [24], spherical to subprolate [25] or spherical [29–31]. The results from the study presented here were similar but much more accurate, because five pollen shape classes were distinguished, of which subprolate and prolate spheroidal pollen shapes dominated.

Kim et al. [22] studied an inter- and intra-specific variation of pollen grains in *S*. *discolor*, *S*. *eriocephala*, *S*. *lucida*, and *S*. *petiolaris*. In their opinion, the pollen grains demonstrated significant interspecific variation, unequal distances between the species, and various degrees of intraspecific variation. Current statistical analyses give similar results.

The presented classification system of analysed *Salix* species was partly based on the current phylogenetic studies, but division into the sections and subsections proposed by Skvortsov [3] was used. Skvortsov’s classification is still the most comprehensive systematic survey for the willow species of Central and Eastern Europe. Clear and well-founded infrageneric grouping and broad species concept, based on extensive field observations and herbarium studies are strong advantages of this traditional system [10]. However, modern studies on the molecular phylogenies partly dispute Skvortsov’s infrageneric classification [32–34, 42–44]. Among others, Wu and co-workers [32] considered the section *Triandrae* should be excluding from subgenus *Salix* s.l. and treated as separate subgenus with solitary species *S*. *triandra*. Taxonomical distinctiveness of *S*. *triandra* was also proved in other studies [42, 43]. The current research did not support so unequivocal evidence. The micromorphological patterns of exine (mainly the character of edges of muri) allowed to separate *S*. *triandra* pollen grains from the other tested species of subgenus *Salix*. At the same time pollen grains of this species were morphologically similar to *S*. *myrtilloides*, *S*. *silesiaca* and *S*. *rosmarinifolia* (subgenus *Chamaetia/Vetrix*). The differences among the pollen grains of all these species were only in visibility of colpus margo. Current analysis of taxonomic relationships also did not confirm a separateness of pollen of *S*. *triandra*. Modern molecular studies undermined Skvortsov’s division into subgenus *Chamaetia* and *Vetrix*. Wagner and colleagues [33] focusing on European species of both subgenera confirmed the monophyly of the *Chamaetia/Vetrix* clade by genomic RAD sequencing markers. They postulated merging subgenus *Chamaetia* and *Vetrix*. The same conclusion was drawn from other molecular studies [32, 43, 44]. Generally, these results were in accordance with current findings. The analysed species did not form groups with the taxonomy. It can be seen when analysing both the qualitative and quantitative characteristics of the pollen grains of the currently studied species. For example, although the pollen of all (five) species of the section *Vetrix* represented the same morphological type, only *S*. *cinerea* and *S*. *aurita* were practically indistinguishable. The morphological similarity of these species was also noted by Sohma [28]. On the other hand, a very close resemblance to *S*. *cinerea* and *S*. *aurita* was observed in pollen grains of *S*. *lapponum* (section *Villosae*) and *S*. *purpurea* (section *Helix*). Instead, the current analysis of quantitative features of pollen grains revealed the distinctiveness of *S*. *cinerea* and *S*. *aurita*. In this regard *S*. *cinerea* was closest to *S*. *starkeana* (Section *Villosae*), *S*. *lapponum* and S. *dasyclados* (section *Vimen*) and *S*. *aurita* to *S*. *viminalis* (section *Vimen*), *S*. *purpurea* and *S*. *repens* (section *Incubaceae*).

In conclusion, the presented study proved that, according to all the analysed biometric pollen features from 24 *Salix* species, and mainly the exine ornamentation, there was no clear relationship between the pollen of the species representing the same subgenera and sections. The pollen traits were most important at species level.
