# Correction: Pollen morphology and variability of Polish native species from genus *Salix* L.

**DOI:** 10.1371/journal.pone.0252253

**Published:** 2021-05-20

**Authors:** Irmina Maciejewska-Rutkowska, Jan Bocianowski, Dorota Wrońska-Pilarek

There are errors in the Funding statement. The correct Funding statement is as follows: This study was funded by the Ministry of Science and Higher Education (Poland) programme "Regional Initiative Excellence" in years 2019–2022, Project No. 005/RID/2018/19.
